# Efficacy of surgery and chemotherapy for stage IV small bowel adenocarcinoma: A population‐based analysis using Surveillance, Epidemiology, and End Result Program database

**DOI:** 10.1002/cam4.3266

**Published:** 2020-08-04

**Authors:** Tongtong Liu, Yunlong Wu, Tao Jiang

**Affiliations:** ^1^ Department of Radiology Beijing Chao‐Yang Hospital Capital Medical University Beijing China; ^2^ Department of Colorectal Surgery National Cancer Center/National Clinical Research Center for Cancer/Cancer Hospital Chinese Academy of Medical Sciences and Peking Union Medical College Beijing China

**Keywords:** chemotherapy, metastasis, small bowel adenocarcinoma, surgery, survival

## Abstract

**Background:**

The role of surgery and chemotherapy for stage IV small bowel adenocarcinoma (SBA) is still confused. The results from previous analyses have been limited by small sample sizes and different treatment regimens.

**Methods:**

Patients with stage IV SBA were identified in the Surveillance, Epidemiology, and End Result Program (SEER) database. Cause‐specific survival (CSS) and overall survival (OS) were calculated with Kaplan‐Meier methods and log‐rank test. Multiple logistic and Cox regression identified covariates associated with treatment options and survival.

**Results:**

1219 eligible patients were involved in this study. The median age was 67 (range, 20‐95) with 655 (53.7%) males and 564 (46.3%) females. Age and primary tumor site were significantly associated with surgery performance, age was also significantly associated with chemotherapy (*P *< .01). To reduce bias, further six subgroups were divided by age (≤65 and >65) and primary tumor site (duodenum, jejunum and ileum). Chemotherapy and surgery conferred a benefit on survival of the whole cohort (the median CSS of different treatment groups were 17, 9, 4, and 1 month respectively, *P *< .001) and most subgroups (83.3%, 5/6). In multivariate analysis, surgery (*P *= .006), and chemotherapy (*P *= .038) are still independent factors of favorable CSS and OS. For patients with surgery (n = 362), radical surgery was not associated with better survival.

**Conclusion:**

For stage IV SBA patients, the present study showed that age and primary tumor site were significantly associated with treatment preference. Surgery and chemotherapy were consistently correlated with favorable survival for the whole cohort or most specific subgroups. However, compared with palliative surgery, significant association was not found in patients with radical surgery with better outcome. More prospective well‐defined cohorts would add knowledge for this rare disease.

## Graphical abstract

Surgery and chemotherapy were prognostic factors consistently for favorable survival. Moreover, for patients who underwent surgery, there is no significant association with better outcome in paitents who underwent radical surgery.

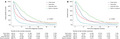

## 1 INTRODUCTION

Small bowel malignancies are rare disease, but the incidence has a rapid growth in recent years with an annual change of more than 3.0 percent.^1^, ^2^ About 11 110 new cases and 1700 deaths of small bowel cancer are expected to be estimated in the United States in 2020, accounting for approximately 3.0% and 1.0% of the digestive system tumors.^3^ About one‐third of small bowel malignancies were adenocarcinoma^1^, ^2^ and the 5‐year overall survival rate was poor (25.0%).^1^ Without specific symptom, more than 30% of SBA patients at diagnosis were stage IV,^1^, ^4^ which partly contributed to the disappointing prognosis.

The approach proposed for colon cancer has been, to some extent, adopted for the treatment of SBA because of the rarity. Radical surgery is the primary recommended and potentially curative treatment for stage I‐III SBA.^5^, ^6^ In a retrospective cohort study of duodenal adenocarcinoma, actuarial 5‐year survival for the curative surgery group (n = 68) was 54%, while only 1 patient's surviving period exceeding 3 years in the palliative procedure group (n = 33).^7^ Moreover, some studies also showed that traditional adjuvant chemotherapy (FOLFOX) might promote the survival rate for advanced SBA with superior response rate and well‐tolerated toxicities.^8^, ^9^ At present, there is still lack of research on treatment for stage IV SBA. Only a few studies have attempted to explore the rational regimen for these patients,^10^, ^11^, ^12^, ^13^ and the results were insecure partly due to the small sample sizes and disparate treatment regimens.

For these reasons, the objective of this study was to further explore if surgery and chemotherapy could bring benefit to SBA patients with distant metastasis, using the population‐based Surveillance, Epidemiology, and End Results (SEER) database.

## 2 MATERIALS AND METHODS

### 2.1 Data source and patient selection

**FIGURE 1:**
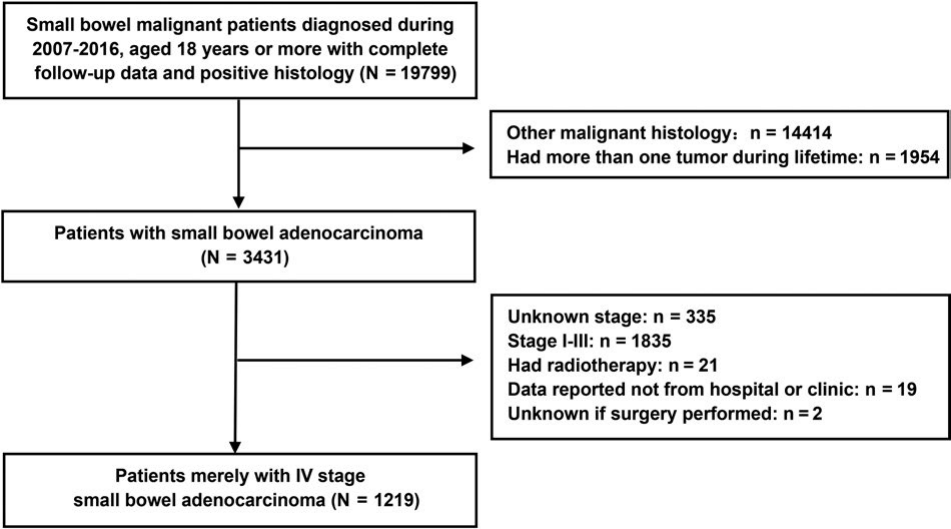
Cohort selection criteria for small‐bowel adenocarcinoma (SBA) from 2007 to 2016 SEER database

This study was based on database^14^ from the SEER program which collects cancer incidence data from population‐based cancer registries covering approximately 34.6 percent of the US population. Small bowel tumors were identified using International Classification of Disease for Oncology, third edition (ICD‐O‐3) sites codes (C17.0‐C17.9). The initial database includes 19 799 patients aged equal or greater than 18 years with complete follow‐up data and positive malignant histology during 2007‐2016. To explore risk factors of survival for SBA, patients had other malignant histology rather than adenocarcinoma or had more than one tumor history during lifetime were excluded. Patients were staged according to the American Joint Committee on Cancer (AJCC) sixth and seventh edition staging classification.^15^, ^16^ Since our study concentrated on SBA with distant metastasis, we excluded patients as stage I‐III or stage unknown. To further decrease the potential bias, we also excluded patients who underwent radiotherapy, with unclear surgery data or those not reported from hospital or clinic. Finally, 1219 patients with stage IV SBA were included in this study for further analysis. (Figure 1).

### 2.2 Variables

We collected these patients’ clinicopathologic features from the SEER database, including diagnosis time, age, sex, race, insurance, tumor site, tumor size, differentiation, T stage, N stage, metastatic site, and data of surgery and chemotherapy. Radical surgery was defined as resection of both the primary and metastatic tumors during the same procedure, and palliative surgery was defined as different levels of resection of primary tumors with or without metastases. Our primary endpoints were cause‐specific survival (CSS: the time from surgery to cancer‐related death or last follow‐up) and overall survival (OS: the time from surgery to death or last follow‐up).

### 2.3 Statistical analysis

Categorical variables are described as frequencies, while continuous variables were tested for normality and are presented as mean and standard deviation (SD) or median. Survival function was estimated by the Kaplan‐Meier method and the differences were evaluated using the log‐rank test. Multivariate logistic regression was used to calculate odds ratios (OR) of factors associated with surgery and chemotherapy. The cutoff value of continuous variables were determined based on the Akaike information criterion (AIC). We also used the Cox proportional hazards regression to identify independent prognostic factors for CSS and OS. In all multivariable analysis, we used a stepwise multiple regression for variable selection based on the AIC. All *P* values reported were two‐tailed. Results were considered statistically significant if *P *< .05. All statistical analyses were performed using R software, version 3.5.3.

## 3 RESULTS

### 3.1 Patients’ characteristics

Patients’ clinical and pathological features were summarized in Table 1. Totally, 1219 stage IV SBA patients were enrolled in this study. The median age was 67 (range 20‐95) with 655 (53.7%) males and 564 (46.3%) females. Duodenum was the most common primary site for SBA (n = 788, 64.6%), followed by jejunum (n = 163, 13.4%) and ileum (n = 93, 7.6%). Metastasis occurred in liver in 421 patients (34.5%), lung in 64 patients (5.3%), bone in 20 (1.6%) patients, and brain in 3 patients (0.2%). There were 468 patients who received chemotherapy (38.4%), 130 patients underwent surgery (10.7%), 232 patients received both chemotherapy and surgery (19.0%), and the remaining part had no treatment (or unknown) (n = 389, 31.9%).

**TABLE 1:** Demographic and clinicopathologic characteristics of the whole cohort (continued)

		Treatment			
	Total (n = 1219)	Chem‐alone (n = 468)	Surg‐alone (n = 130)	Surg‐Chem (n = 232)	None (n = 389)
Diagnose time (y)					
2007‐2011	535 (43.9%)	187 (40.0%)	71 (54.6%)	98 (42.2%)	179 (46.0%)
2012‐2016	684 (56.1%)	281 (60.0%)	59 (45.4%)	134 (57.8%)	210 (54.0%)
Age (y)					
Median [Min, Max]	67[20.0, 95.0]	65.5[21.0, 92.0]	68.0[35.0, 93.0]	59 [20.0, 91.0]	73[28.0, 95.0]
Sex					
Female	564 (46.3%)	220 (47.0%)	70 (53.8%)	101 (43.5%)	173 (44.5%)
Male	655 (53.7%)	248 (53.0%)	60 (46.2%)	131 (56.5%)	216 (55.5%)
Race					
American Indialaska Native	4 (0.3%)	1 (0.2%)	1 (0.8%)	1 (0.4%)	1 (0.3%)
Asian or Pacific Islander	85 (7.0%)	35 (7.5%)	7 (5.4%)	17 (7.3%)	26 (6.7%)
Black	269 (22.1%)	107 (22.9%)	31 (23.8%)	57 (24.6%)	74 (19.0%)
White	859 (70.5%)	325 (69.4%)	91 (70.0%)	155 (66.8%)	288 (74.0%)
Missing	2 (0.2%)	0 (0%)	0 (0%)	2 (0.9%)	0 (0%)
Insurance					
Insured	1173 (96.2%)	450 (96.2%)	126 (96.9%)	225 (97.0%)	372 (95.6%)
Uninsured	46 (3.8%)	18 (3.8%)	4 (3.1%)	7 (3.0%)	17 (4.4%)
Site					
Duodenum	788 (64.6%)	378 (80.8%)	31 (23.8%)	40 (17.2%)	339 (87.1%)
Ileum	93 (7.6%)	6 (1.3%)	34 (26.2%)	49 (21.1%)	4 (1.0%)
Jejunum	163 (13.4%)	29 (6.2%)	30 (23.1%)	87 (37.5%)	17 (4.4%)
Missing	175 (14.4%)	55 (11.8%)	35 (26.9%)	56 (24.1%)	29 (7.5%)
Tumor size(mm)					
Mean (SD)	49.3 (± 67.8)	54.7 (± 94.7)	44.2 (± 24.0)	43.3 (± 26.8)	55.7 (± 89.2)
Missing	621 (50.9%)	293 (62.6%)	29 (22.3%)	32 (13.8%)	267 (68.6%)
Differentiation					
Well	428 (35.1%)	159 (34.0%)	54 (41.5%)	83 (35.8%)	132 (33.9%)
Moderate	250 (20.5%)	102 (21.8%)	25 (19.2%)	51 (22.0%)	72 (18.5%)
Poor	156 (12.8%)	66 (14.1%)	19 (14.6%)	26 (11.2%)	45 (11.6%)
Undifferentiated	27 (2.2%)	11 (2.4%)	2 (1.5%)	4 (1.7%)	10 (2.6%)
Missing	358 (29.4%)	130 (27.8%)	30 (23.1%)	68 (29.3%)	130 (33.4%)
T stage					
T0‐2	167 (13.7%)	77 (16.5%)	5 (3.8%)	11 (4.7%)	74 (19.0%)
T3‐4	630 (51.7%)	189 (40.4%)	118 (90.8%)	209 (90.1%)	114 (29.3%)
Missing	422 (34.6%)	202 (43.2%)	7 (5.4%)	12 (5.2%)	201 (51.7%)
N stage					
Negative	508 (41.7%)	199 (42.5%)	45 (34.6%)	68 (29.3%)	196 (50.4%)
Positive	496 (40.7%)	171 (36.5%)	78 (60.0%)	150 (64.7%)	97 (24.9%)
Missing	215 (17.6%)	98 (20.9%)	7 (5.4%)	14 (6.0%)	96 (24.7%)
Metastasis site					
Bone	20 (1.6%)	9 (1.9%)	2 (1.5%)	3 (1.3%)	6 (1.5%)
Brain	3 (0.2%)	2 (0.4%)	1 (0.8%)	0 (0%)	0 (0%)
Liver	421 (34.5%)	199 (42.5%)	26 (20.0%)	53 (22.8%)	143 (36.8%)
Lung	64 (5.3%)	29 (6.2%)	7 (5.4%)	5 (2.2%)	23 (5.9%)
Other or multiple	386 (31.7%)	132 (28.2%)	49 (37.7%)	112 (48.3%)	93 (23.9%)
Missing	325 (26.7%)	97 (20.7%)	45 (34.6%)	59 (25.4%)	124 (31.9%)
Surgery					
No	857 (70.3%)	468 (100%)	0 (0%)	0 (0%)	389 (100%)
Yes	362 (29.7%)	0 (0%)	130 (100%)	232 (100%)	0 (0%)
Chemotherapy					
No	519 (42.6%)	0 (0%)	130 (100%)	0 (0%)	389 (100%)
Yes	700 (57.4%)	468 (100%)	0 (0%)	232 (100%)	0 (0%)

Abbreviations: Chem‐alone, chemotherapy alone; None, without any treatment; Surg‐alone, surgery alone; Surg‐Chem, surgery with chemotherapy.

### 3.2 Factors associated with chemotherapy and surgery

Given that patients who received surgery and chemotherapy always had better physical condition or less tumor burden, we then explored the clinical factors which were associated with treatment preference for stage IV SBA patients determined by multiple logistic regression. As shown in Table 2, younger patients with distal small bowel tumors (ileum and jejunum) were more likely to have surgery (*P *< .01). For chemotherapy, age was the only significant factor (*P *< .001), in other words, elder patients tend to not receive chemotherapy. Based on AIC value, 61 and 71 years of age were cutoff values for surgery and chemotherapy preference respectively (Figure S1). As the median age of the whole cohort was 67 years old (Table 1), we finally chose 65 years as the cut‐off value for further analysis.

**TABLE 2:** Multivariate analysis of logistic regression

	Surgery			Chemotherapy		
	OR	95%Cl	*P* value	OR	95%Cl	*P* value
Age	0.97	(0.94‐0.99)	.002	0.94	(0.93‐0.96)	<.001
Site						
Duodenum	reference	reference		—	—	—
Ileum	553.29	(111.64‐10 083.04)	<.001	—	—	—
Jejunum	17.76	(9.95‐32.68)	<.001	—	—	—
Metastasis site						
Bone	reference	reference		—	—	—
Brain	18.15	(0.43 −912.34)	.111	—	—	—
Liver	1.4	(0.21‐17.6)	.766	—	—	—
Lung	0.73	(0.07‐11.45)	.806	—	—	—
Other or multiple	3.58	(0.54‐45.18)	.254	—	—	—
Insurance						
Insured	reference	reference		—	—	—
Uninsured	0.01	(0.00‐0.18)	.004	—	—	—

Abbreviations: CI, confidence interval; OR, odds ratio.

### 3.3 Effect of chemotherapy and surgery on survival in subgroups and multivariate analysis

**FIGURE 2:**
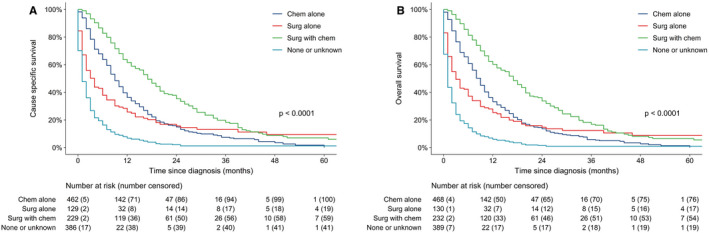
CSS (A) and OS (B) between different treatment regimens for the whole stage IV SBA cohort

The median follow‐up period was only 5 months (range, 0‐115 months) due to the poor outcome. Survival was examined for patients with different treatments. CSS of patients who received surgery and chemotherapy was better than patients with chemotherapy or surgery alone, the outcome of the patients who did not receive any treatment was the worst (the median CSS were 17,9, 4, and 1 month respectively, Log Rank *P* < .001). Similar results were shown for OS (Figure 2A,B).

**FIGURE 3:**
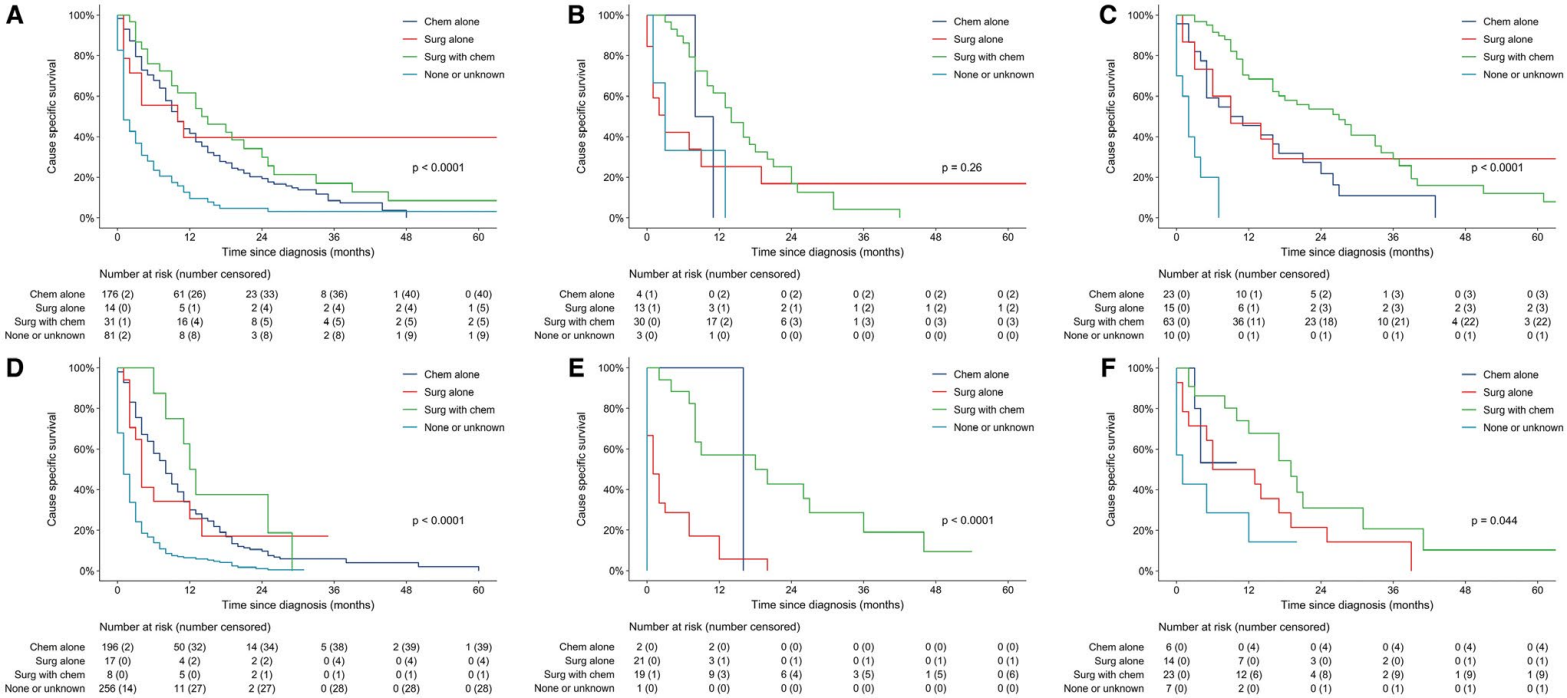
CSS between different treatment regimens for subgroups of young‐duodenum(A), young‐ileum (B), young‐jejunum (C), old‐duodenum (D), old‐ileum (E), and old‐jejunum (F)

**FIGURE 4:**
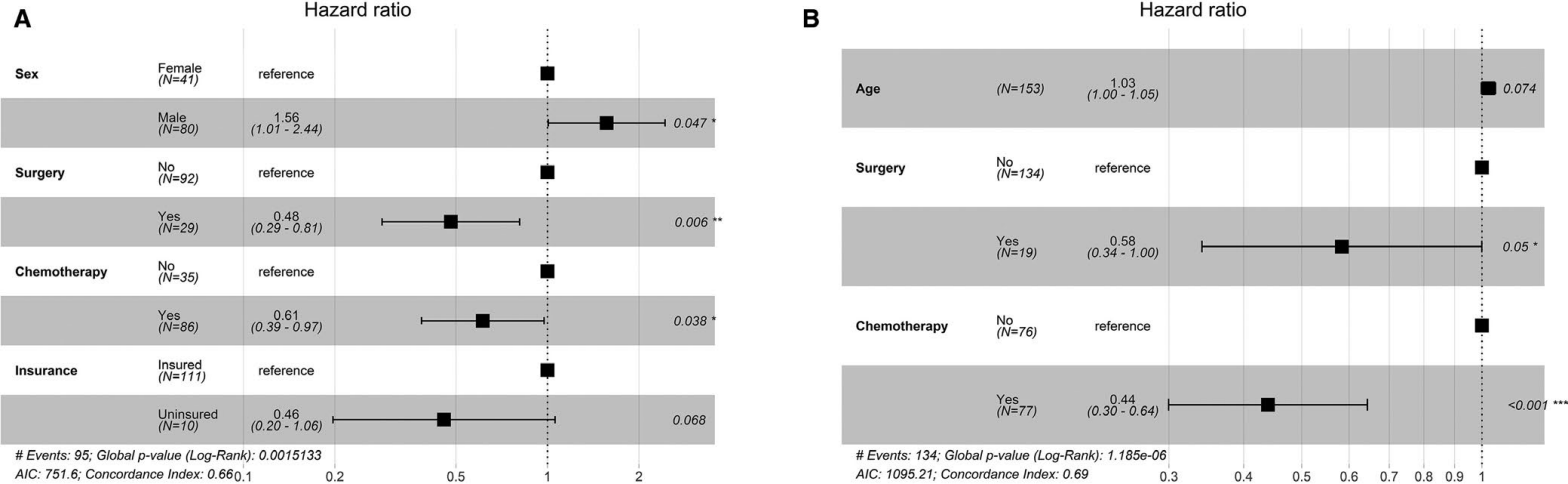
Forest plot of multivariate COX analysis of CSS for subgroups of old‐duodenum(A) and young‐duodenum (D)

To further investigate the benefit of surgery and chemotherapy, we divided all the patients into six subsets by age (≤65 and > 65 years old) and primary tumor site (duodenum, ileum, and jejunum). In majority of the subsets (83.3%,5/6), surgery and chemotherapy were still significantly associated with better CSS (Figure 3A‐F) and OS (Figure S2A‐F) on univariate analysis. Furthermore, in the two largest subsets (duodenum and age > 65 or ≤ 65), multivariate analysis revealed that surgery and chemotherapy were independent prognostic factors for better CSS (Figure 4A‐B) and OS (Figure S3A‐B).

With respect to the data shown above, patients with surgery would have a better outcome, it was rational to further explore if surgery type (radical or not radical) was associated with survival. Among 362 patients who underwent surgery, radical surgery was performed in 62 patients (17.1%). Patients with radical surgery had a significant better CSS and OS than the palliative group on univariate analysis (*P* = .018) (Figure S4A,B). However, in multivariate analysis, chemotherapy, age, and tumor grade were independent prognostic factors, but not radical surgery (Figure S5A,B).

## 4 DISCUSSION

This large, population‐based study revealed meaningful results for the treatment of stage IV SBA patients. Elder patients with duodenum as the primary site of tumor location were less likely to receive surgery and were also prone to not receive chemotherapy. In majority of the subgroups and multivariate analysis, surgery and chemotherapy were constantly significant prognostic factors for prolonged survival of patients with stage IV SBA. Moreover, radical surgery was associated with better survival in univariate analysis but non‐significant in multivariate analysis.

Surgery is the only potential curative method for stage IV colorectal cancer (CRC) patients. The 5‐year OS of patients with resectable liver metastasis CRC could reach to 58%.^17^ For stage IV SBA patients without any treatment, the OS were extremely poor, ranging from 2 to 5.9 months^5^, ^18^, ^19^ and the studies to explore the efficacy of surgery for stage IV SBA were scarce. 152 stage IV SBA patients with peritoneal metastases were enrolled in a restrospective study in 2018, prolonged survival have been archived using combined treatment strategy of cytoreductive surgery plus hyperthermic intraperitoneal chemotherapy (median OS was 32 months with survival rates of 46.4% at 3 years). Multivariate analysis revealed that absence of lymph node metastasis, well‐differentiated tumor, and peritoneal cancer index of 15 or lower were factors independently associated with improved OS.^11^ Recently, another retrospective research enrolled 34 stage IV SBA patients with surgery, and the median OS and RFS were 28.6 and 18.7 months respectively.^12^ Consistent with former studies,^11^, ^12^ we found that surgery was an independent factor associated with better survival for stage IV SBA.

Compared with other surgery procedures, radical surgery was associated with better survival in univariate analysis, but not in multivariate analysis. There may be some explanations for this incredible result; first of all, there were 62 patients who underwent radical surgery in this cohort and the lack of the effect of radical surgery in multivariate analysis may be due to underpowered number of patients; second, stage IV SBA is highly malignant and most patients would have recurrence soon after the so‐called radical surgery; third, radical surgeries always mean large range of resection with high rate of complications and mortality; and at last, palliative surgery which resect primary tumor would avoid the risk of obstruction, hemorrhage, or perforation. In 2015, a large‐scale retrospective study included 1982 CRC patients with unresectable metastasis revealed that, even palliative primary tumor resection significantly improved CSS among the whole patient population.^20^ And for metastatic SBA, several research studies also found that resection of primary tumor is one of the independent factors of OS.^19^, ^21^, ^22^

In our analysis, independent prognostic factors for these patients with surgery were age, chemotherapy, and tumor grade instead of different surgery procedures. Other clinicopathologic factors associated with patient survival have been explored in some studies, such as performance status score,^8^, ^18^, ^23^, ^24^, ^25^ poor differentiation,^11^, ^12^, ^24^ and carcinoembryonic antigen (CEA).^8^, ^18^, ^25^Moreover, Rompteaux P et al also found the overall survival of patients who underwent resection of metastases with poorly differentiated tumor verged on the patients merely with palliative chemotherapy, indicating that only patients with well or moderate differentiated tumors might benefit from resection of metastases.^12^Although prognostic factors for survival do not equal to predictive factors for treatment effect, these results still remind us that above prognostic factors maybe helpful to further select specific subgroups that will have benefit from specific therapy, especially for CEA and performance status score which are available before surgery. More researches are needed to further explore the predictive factors for treatment effect in patients with stage IV SBA.

Besides, chemotherapy also significantly associated with better survival in our analysis, no matter surgery was performed or not. For stage IV or unresectable SBA patients, several analyses found that palliative chemotherapy could prolong survival compared with best supportive care or without treatment.^5^, ^18^, ^19^, ^24^, ^26^ Among different chemotherapy regimens, combination chemotherapy including platinum and 5‐FU are primarily recommended as yet, with well tolerance and better OS.^6^, ^8^, ^9^, ^10^, ^11^, ^21^, ^22^, ^25^, ^26^, ^27^ As distinct genomic profiling of SBA was revealed compared with CRC and gastric carcinoma,^28^ it seems unreasonable that the approach proposed for CRC or gastric carcinoma was completely adopted for the treatment of SBA. In 2016, a 15‐year retrospective study from large registry revealed that chemotherapy was one of the favorable prognostic factors in metastatic disease (10 months vs 3 months), however, the median OS did not increased in these patients along with time,^1^ which partly indicated that there were few remarkable improvements of therapy and the efficacy of common regimens was still poor. Recently, some phase II prospective studies began to explore the safety and efficacy of new regimens, such as bevacizumab combined with capecitabine and oxaliplatin,^29^ and panitumumab in RAS wild‐type patients,^13^ but the results need to be further explored with more large‐scale trials. It is a pity that detailed chemotherapy information is unavailable from SEER database, but on the whole, survival period of patients with stage IV SAB could be extended by using chemotherapy.

This study has several limitations. First, this was a retrospective analysis and the potential heterogeneity of enrolled patients would influence the statistical analysis. Second, the lack of data for some recognized prognostic parameters, such as performance status and chemotherapy regimen restrained our further analysis. Finally, we excluded patients with missing data that might increase the bias.

In conclusion, this study was performed to examine the influence factors and the efficacy of surgery and chemotherapy for stage IV SBA using large population‐based SEER database. We found that elder patients with duodenum tumors were unlikely to receive any treatment. However, in majority of the subgroups and multivariate analysis, surgery and chemotherapy were prognostic factors required for favorable survival. For patients with surgery, surgery type (radical or not) was not found in significant association with the outcome. Clinically, reliable treatment regimens should be personalized according to patients’ complex conditions, and prospective well‐defined cohorts are extremely needed to further explore more effective and precise therapies for different subgroups of SBA patients.

## CONFLICTS OF INTEREST

The authors have no conflict of interest to declare.

## AUTHOR CONTRIBUTIONS

Tongtong Liu: Conception and design, collection and assembly of the data, writing, and editing.

Yunlong Wu: Conception and design, data analysis and interpretation.

Tao Jiang: Conception and design, supervision, editing, and review.

## Supporting information

Fig S1

Fig S2

Fig S3

Fig S4

Fig S5

## Data Availability

The data that support the findings of this study are openly available in [Surveillance, Epidemiology, and End Results (SEER) Program] at [www.seer.cancer.gov], reference number [14].
