# Supplementary figures and images for: Efficacy of surgery and chemotherapy for stage IV small bowel adenocarcinoma: A population‐based analysis using Surveillance, Epidemiology, and End Result Program database
Source: Cancer Med. 2020 Aug 4;9(18):6638–45. doi: 10.1002/cam4.3266 (PMC7520278; doi:10.1002/cam4.3266)

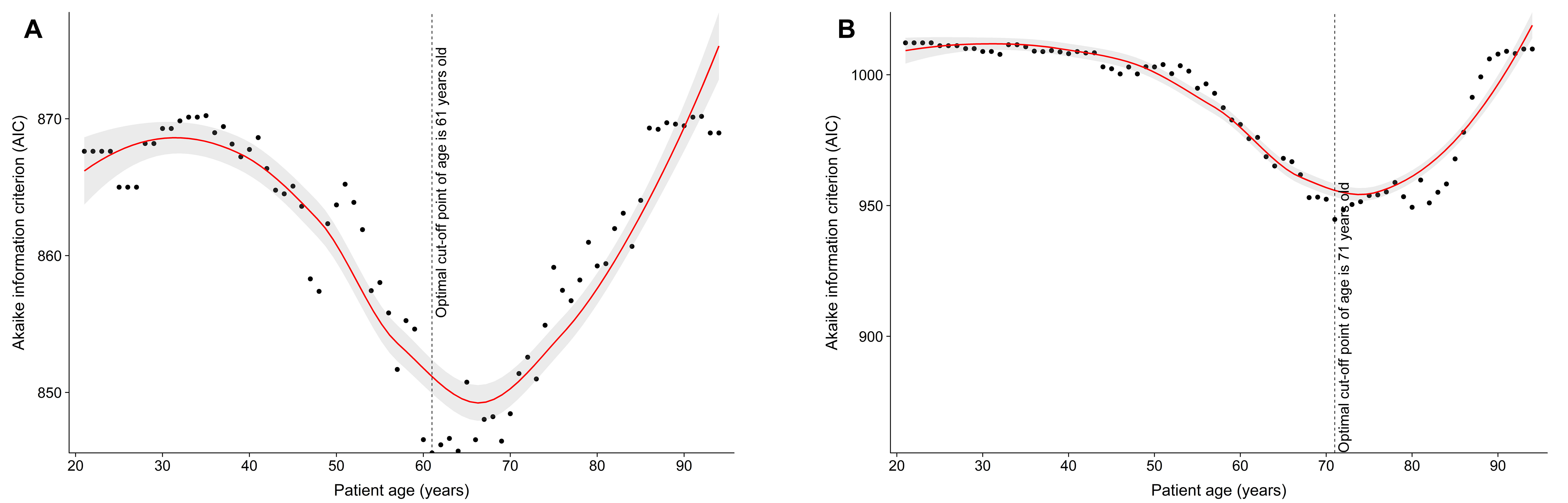

Supplement: Supplementary file 1 — Fig S1 [file CAM4-9-6638-s001.jpg]

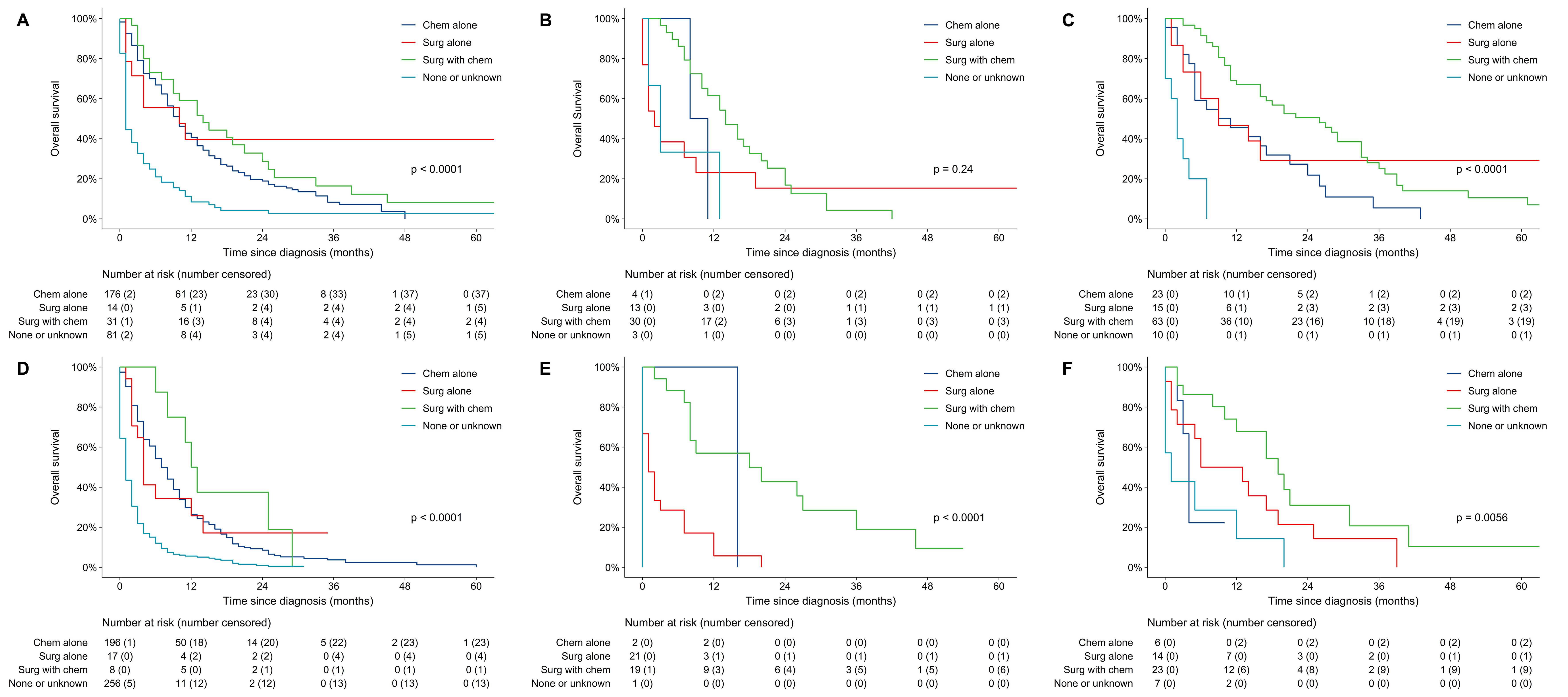

Supplement: Supplementary file 2 — Fig S2 [file CAM4-9-6638-s002.jpg]

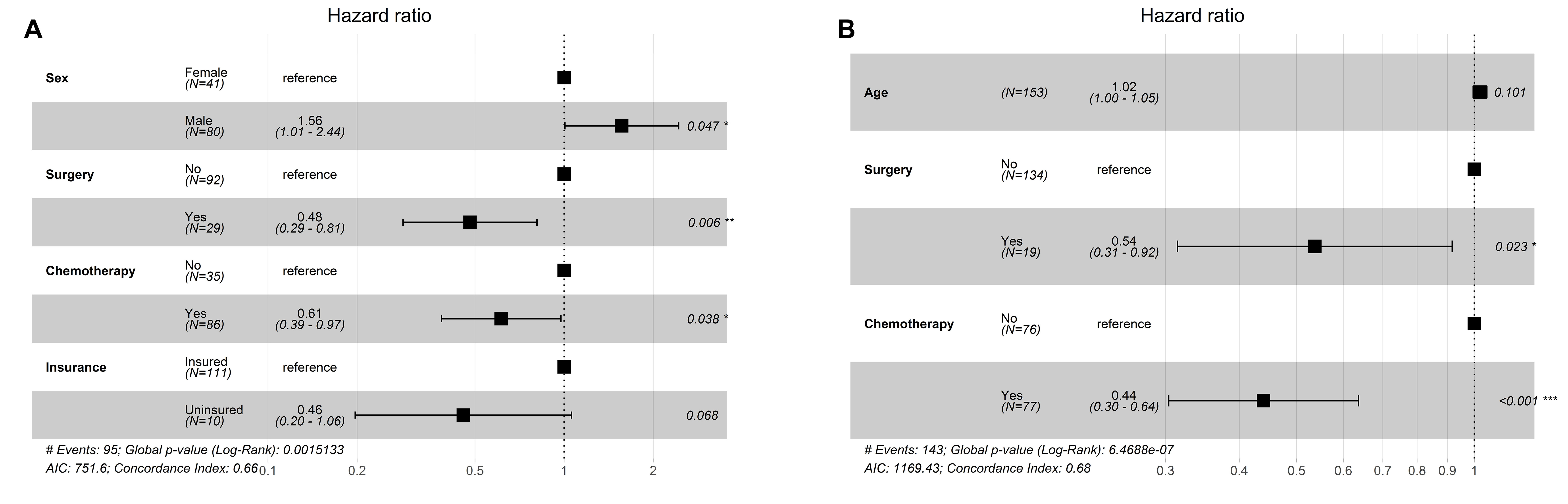

Supplement: Supplementary file 3 — Fig S3 [file CAM4-9-6638-s003.jpg]

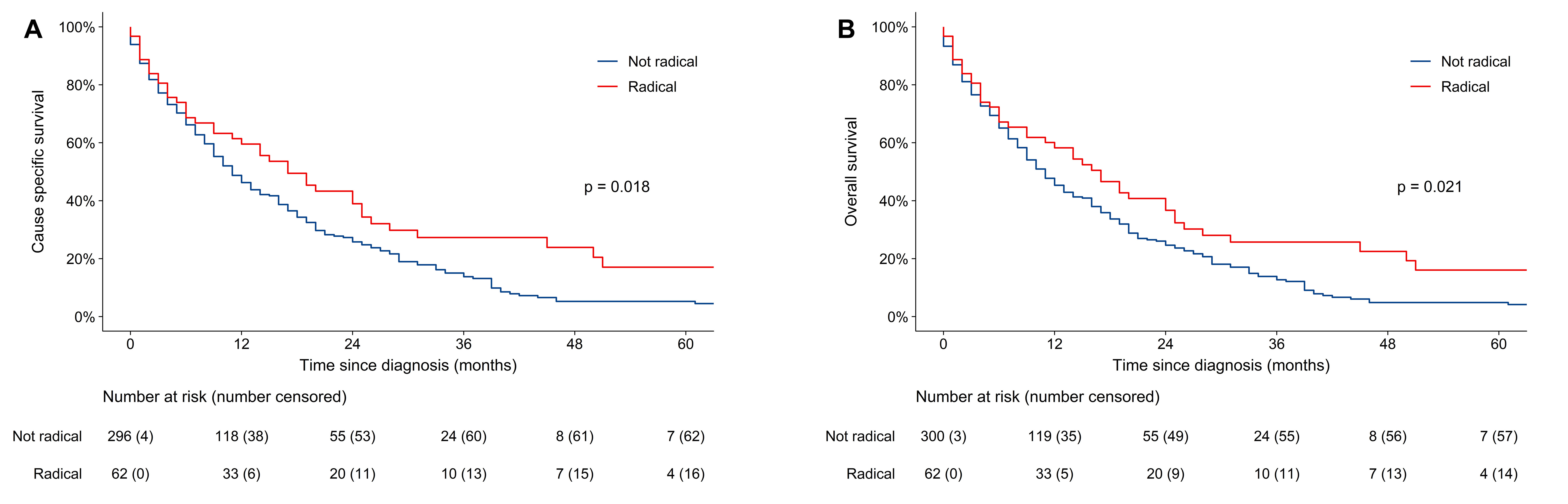

Supplement: Supplementary file 4 — Fig S4 [file CAM4-9-6638-s004.jpg]

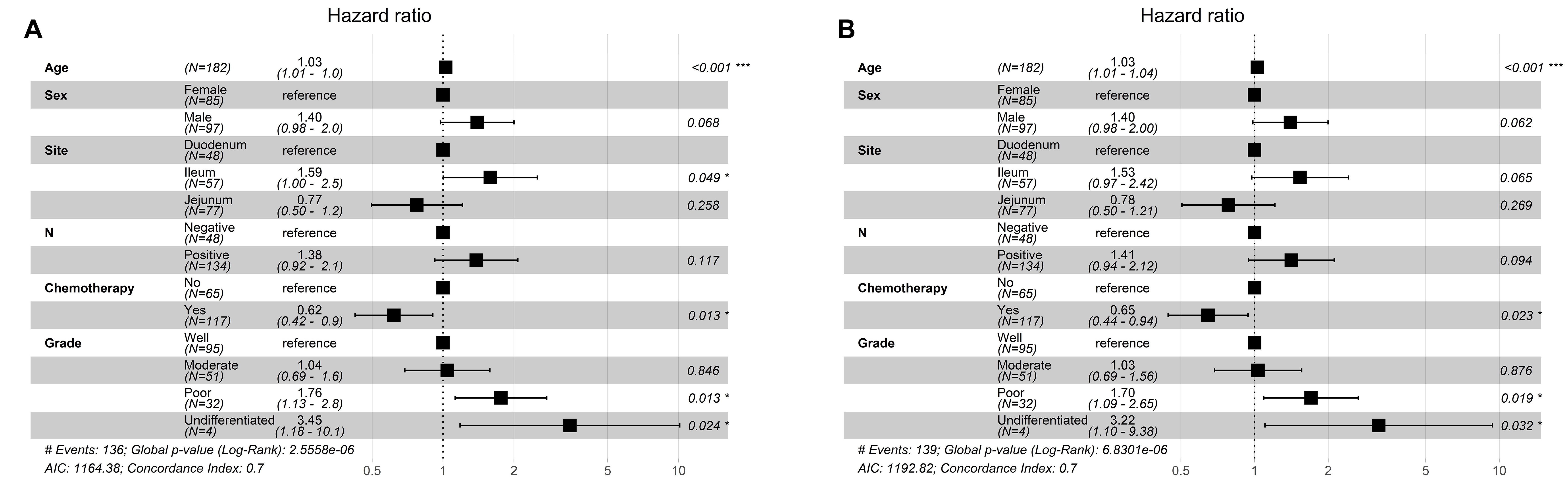

Supplement: Supplementary file 5 — Fig S5 [file CAM4-9-6638-s005.jpg]
